# Photothermal conditioning of platelet-rich plasma: mechanisms and emerging applications in hair regeneration

**DOI:** 10.1093/skinhd/vzaf089

**Published:** 2025-12-22

**Authors:** Ilknur Nihal Ardic, Nurittin Ardic

**Affiliations:** Este Medical Group, Hair and Skin Clinic, Birmingham, UK; Med-International UK Health Agency Ltd, Leicestershire, UK

## Abstract

Photothermal conditioning of platelet-rich plasma (PRP) is an emerging innovation in regenerative medicine, particularly in the field of hair restoration. By using controlled electromagnetic energy, this technique modulates the biological activity of PRP, potentially enhancing the release of growth factor, production of exosomes and communication between cells. This review aims to explore the mechanisms behind photothermal PRP, summarize current preclinical and clinical findings, and evaluate its applications in hair regeneration. A comprehensive literature review was conducted across multiple databases to evaluate mechanistic data, clinical outcomes and technology platforms. Preliminary evidence suggests that photothermal conditioning enhances the bioactivity of PRP, leading to potential increases in hair density and follicular regeneration. Additionally, it may help reduce inflammation and prolong the anagen phase of the hair cycle. However, there is currently a lack of standard protocols, dosage regimens and clear understanding of the mechanisms involved. Despite these limitations, photothermal PRP shows promise as an adjunctive or alternative treatment for androgenic and inflammatory alopecia. Future clinical studies with standardized protocols and larger cohorts are necessary to confirm its efficacy, optimize treatment parameters and ensure long-term safety.

## Teaser

Photothermal conditioning represents a novel approach to enhance the therapeutic potential of platelet-rich plasma in hair regeneration. This review provides insights into its growing importance in regenerative dermatology by highlighting recent advances in technology, underlying biological mechanisms and clinical applications.

What is already known about this topic?

- Photothermal conditioning of platelet-rich plasma (PRP) is a novel approach.
- This approach enhances growth factor release and exosome production, potentially improving outcomes in regenerative therapies.
- Its application in hair restoration is emerging but has been understudied, with limited standardization and mechanistic clarity.

What does this study add?

- This review summarizes the current evidence on photothermal PRP for hair regeneration, detailing its biological mechanisms, early clinical results and potential advantages over conventional PRP.
- It highlights gaps in standardization and suggests future research directions.

Hair loss is a common cosmetic concern for millions of people worldwide, significantly affecting self-esteem, social interaction and psychological wellbeing. Traditional treatment methods for hair loss include pharmacological methods such as oral finasteride, topical minoxidil or surgical hair transplantation.^1^ The aetiology of hair loss plays an important role in the selection of pharmacological drugs. Finasteride or minoxidil are preferred for androgenic alopecia, while corticosteroids are preferred for alopecia areata.^2^ However, these methods have limitations such as variable effectiveness and additional invasiveness for hair transplantation. Another method utilizes nonpharmaceutical low-level laser therapy.^3^

The pathophysiology of hair loss involves complex interactions between factors such as genetic predisposition, hormonal influences and inflammatory processes. Understanding the growing process and pathophysiology of hair loss is crucial for developing and selecting the most appropriate targeted therapeutics that can interrupt or reverse this pathological process. The average hair growth cycle consists of four phases: anagen (growth), catagen (involution), telogen (resting) and exogen (hair loss). Those in the anagen phase determine hair length and make up 80–90% of the hair. Less than 5% are in the catagen phase while the rest are in the telogen phase. In the natural exogenous phase, approximately 100 hairs are shed per day.^4^ In androgenetic alopecia, dihydrotestosterone binds to androgen receptors in hair follicles, leading to shortening of the anagen phase and lengthening of the telogen phase. This process involves the formation of increasingly thinner and shorter hair shafts and the miniaturization of the follicles, which eventually results in hair loss.^5^

Regenerative medicine applications have recently become an important approach as an adjunctive or alternative to surgery or pharmacological treatment. As a regenerative medicine application, platelet-rich plasma (PRP), which is administered via a microneedling procedure, is often applied. Another regenerative medicine application is using adipose-derived stem cells (ADSCs). Both are autologous biomaterial and play important roles in tissue regeneration and hair regrowth.^6,7^ These biomaterials, which are completely natural, contain numerous growth factors, cytokines and other bioactive molecules that can accelerate wound healing and tissue repair. As a minimally invasive treatment option, these applications are gaining significant interest for hair restoration.^8^

The conventional regenerative methods also have limitations such as biological variability, loss of bioactive mol­ecules and differences in clinical outcomes. Moreover, their preparation and administration methods are not standardized.^8^ These limitations have led researchers to explore development strategies where photothermal conditioning represents a new approach to optimizing the therapeutic potential of PRP.^7^

Despite encouraging preliminary results, photothermal conditioning of PRP remains an evolving field. Mechanistic studies are ongoing and universally accepted standards for protocols, dosage and outcome measurements are lacking. This review therefore aims to critically appraise the current evidence while acknowledging the current gaps in mechanistic clarity and clinical validation.

## Materials and methods

### Literature search strategy

A comprehensive literature review was conducted using electronic databases, including PubMed/MEDLINE, Scopus and Google Scholar. The search strategy included keywords such as ‘platelet-rich plasma’, ‘PRP’, ‘hair loss’, ‘androgenetic alopecia’, ‘hair regeneration’, ‘photothermal conditioning’, ‘photobiomodulation’, ‘exosomes’ and ‘growth factors’.

### Inclusion and exclusion criteria

Peer-reviewed articles published in English, studies investigating PRP therapy for hair loss treatment, research on photothermal conditioning or photobiomodulation of PRP, clinical studies, case series and experimental studies, as well as articles examining exosome release and cellular mechanisms were included in the study. Non-English publications, conference abstracts without full text and studies focusing solely on nonhair regenerative applications were excluded.

## Results

This section summarizes the key features of the basic biological mechanisms, clinical outcomes, technological innovations and comparative benefits of conventional PRP and photothermal enhancement for hair regeneration based on the reviewed literature to provide a comprehensive understanding of the current evidence base and practical implications.

### Conventional platelet-rich plasma applications in hair regeneration

#### Mechanisms of action and biological foundation

PRP therapy involves collecting autologous blood, concentrating platelets through centrifugation and reinjecting the separated plasma into areas of the scalp with thinning hair.^9^ It is stated that the role of high platelet concentration, which is usually three to five times above basal levels, containing numerous bioactive molecules necessary for tissue regeneration and repair processes, is important in the therapeutic effectiveness of PRP.^10^ Studies have shown that platelets not only impact the homeostatic system, but also affect the inflammatory system, angiogenesis, stem cell induction and cell proliferation through the release of various key regenerative growth factors and cytokines, such as platelet-­derived growth factor (PDGF), vascular endothelial growth factor (VEGF), transforming growth factor-beta (TGF-β) and ­insulin-like growth factor-1 (IGF-1).^7,11^ These growth factors work synergistically in multiple ways to support hair follicle regeneration. PDGF is an important stimulator of dermal papilla cell proliferation and migration, which are fundamental processes for hair follicle development and maintenance. VEGF promotes local angiogenesis to ensure adequate nutrient and oxygen supply for optimal follicular function. IGF-1 stimulates keratinocyte proliferation and delays apoptosis in hair follicle cells, while TGF-β regulates the hair cycle by affecting the transition between growth phases.^10^ In Table 1, conventional PRP and photothermal-assisted PRP are compared in terms of biological response, clinical outcomes and procedural standards.

**Table 1:** Comparison of conventional platelet-rich plasma (PRP) and photothermal-assisted PRP

Component	Conventional PRP	Photothermal conditioned PRP	Reference
Activation technique	Chemical (e.g. calcium chloride, thrombin) or physical activation	Photobiomodulation + thermal stimulation	^12^
Bioactive compound secretion	Baseline granule secretion	2- to 3-fold increase in growth factor levels	^13^
Exosome production	Baseline or limited production	Enhancement up to 5-fold	^14^
Immunogenicity risk	Possible with activating compounds	Negligible (fully autologous, chemical-free)	^12^
Protocol standardization	Heterogenous preparation protocols	Precise temperature/light control (e.g. MCT system)	^15^
Clinical effectiveness	Moderate therapeutic improvement	Accelerated response, increased hair density, sustainable therapeutic outcomes	^12^
Patient satisfaction	∼70–80%	>80%	^16^
Regulatory clarity	Broadly recognized	Improved protocol still meets autologous/minimally manipulated criteria	^15^

MCT, Meta Cell Technology.

#### Clinical evidence and outcomes

PRP can be used as a new treatment option for alopecia, either as monotherapy or as an adjunct to conventional therapy or hair transplantation, with no significant side effects observed. As PRP is derived from the patient’s own blood, the PRP is injected into the scalp to stimulate the release of growth factors that promote hair follicle activity.^8^ Improvements in hair density and thickness following PRP treatment have been observed in early studies. The study by Khatu *et al*. reported an increase in average hair density from 71 to 93 hairs per cm² after PRP treatment.^16^ Another study noted significant increases in hair count and hair diameter.^17^ Meta-analyses of PRP studies have shown consistent improvements in hair density, although with ­moderate-to-large differences in different patient populations. The meta-analyses showed that PRP treatment increases hair density and hair thickness for men and women. The probability of occurrence of clinical effects among the entire study population was reported as follows: improvement in hair density (patient’s rating: 64%; clinician’s rating: 46%), thickness (38% and 45%, respectively), quality (46% and 54%, respectively), sheen/luster (27% and 21%, respectively), new hair growth (57% and 68%, respectively), less hair loss (48% and 20%, respectively) and negative effects (0% and 0%, respectively).^18^ In women, PRP showed a standardized mean difference (SMD) of +2.98 [95% confidence interval (CI) 1.10–4.85] in terminal hair density vs. controls.^19^ A larger meta-analysis in mixed cohorts reported an SMD of approximately +1.21 (95% CI 0.59–1.82) for hair density vs. baseline.^20^ Patient satisfaction rates are generally over 80%, with more favourable results observed in patients with early-stage androgenic alopecia.^21^ Long-term follow-up studies have shown continued improvements in hair parameters up to 12 months after treatment. However, to maintain optimum results, clinical practice guidelines generally recommend maintenance sessions every 6–12 months.^18^

#### Limitations and challenges

Despite its potential, there is considerable variability in PRP preparation methods, injection techniques and treatment protocols among studies. This lack of standardization in PRP preparation, such as activation methods and centrifugation techniques, has resulted in different platelet concentrations and cellular compositions. For example, some studies use double-spin techniques, while others use single-spin techniques.^22,23^ Along with the lack of standardization, factors such as patient-related biological variability, and the limited stability and short half-life of growth factors in conventional PRP may diminish therapeutic efficacy.^8,24^ Individual differences such as age and sex contribute to the heterogeneity in platelet function, growth factor content and response to treatment. This ultimately leads to the heterogeneity of clinical outcomes. Additionally, the rapid degradation of growth factors and other bioactive molecules in traditional PRP preparations limits their therapeutic window and may require more frequent treatment sessions.^25^ In addition to photothermal conditioning, several other methods have been proposed to increase PRP efficacy. For example, chemical activation using calcium gluconate or thrombin has been widely used to induce platelet degranulation. However, the use of these exogenous additives is not without limitations. Sodium citrate can induce platelet aggregation, making accurate counts difficult and affecting results. Xenogeneic thrombin can trigger immunogenic responses, and these additives may require additional equipment or kits, increasing procedural complexity and cost. From a clinical and regulatory perspective, PRP systems that reduce or eliminate the need for extraneous activators, such as photothermal or temperature-based activation methods, are increasingly preferred due to their autologous and minimally manipulated nature.^13^

### Role of exosomes in hair regeneration

#### Biological properties and functions

Recent studies have highlighted that exosomes released by activated platelets and other cells may play a central role in mediating the regenerative effects of PRP. Exosomes are nanometer-scale extracellular vesicles (30–150 nm) secreted by various cell types and play an important role in cell–cell communication through bioactive molecules such as proteins, lipids and microRNAs (miRNAs).^11^ These ­membrane-bound vesicles represent an advanced intercellular communication system that can transfer functional molecules between cells, influencing recipient cell behaviour and function.^10^ In addition to being autologous, exosomes often reflect the characteristics of the cell from which they are secreted.^26^ For example, platelet-derived exosomes secrete exosomes loaded with growth factors such as VEGF, PDGF and TGF-β.^6^ Mesenchymal stem cell-derived exosomes are rich in bioactive molecules, such as lipids, mRNA and ­miRNAs, which regulate immune responses, promote angiogenesis and reduce inflammation.^27^ Depending on the cellular source, activation status and environmental conditions, the cargo composition and function of exosomes vary significantly. Platelet-derived exosomes contain not only growth factors, but also adhesion molecules, coagulation factors, and immunomodulatory proteins that contribute to their regenerative potential. Functional differences can be observed in the exosomal cargo from activated platelets depending on the health or disease state. The lipid composition of exosomal membranes also plays an important role in their stability, cellular uptake and biological activity.^23,28^

#### Therapeutic applications in hair loss

Applications of exosome delivery in hair loss have shown effectiveness in promoting dermal papilla cell proliferation, enhancing hair follicle stem cell activation and modulating inflammatory responses. Exosomes, derived from PRP or ADSCs, can improve therapeutic outcomes by regulating angiogenesis, inflammation and follicular regeneration.^29,30^ The ability of exosomes to cross cellular barriers and deliver cargo directly to target cells makes them crucial for therapeutic use.

However, natural exosome production from traditional PRP is limited, necessitating new production strategies. A preclinical study by Rajendran et al. demonstrated that mesenchymal stem cell-derived exosomes increased dermal papilla cell viability and enhanced hair shaft elongation.^31^ These findings suggest that exosome-mediated therapy could be a more targeted and effective treatment for hair loss. Recent studies have identified specific miRNA profiles within exosomes that are linked to hair regeneration outcomes. The miR-31, miR-125b and miR-200 families play a role in regulating hair follicle stem cells and could be used as biomarkers for treatment response. These findings open up a possibility for personalized treatment strategies based on individual exosome miRNA profiling.^32,33^ The autologous nature of these therapies reduces the risk of immune reactions and disease transmission, making them an appealing option for clinical use. Nonetheless, regulatory authorities are still evaluating the safety profiles of autologous-derived exosome-based therapies.

### Photothermal conditioning: mechanism and rationale

#### Scientific principles and molecular mechanisms

Photothermal conditioning involves the application of light at specific wavelengths (e.g. red or near-infrared) and mild heat to biological tissues or products like PRP. This process activates opsin receptors and transient receptor potential ion channels, triggering downstream G-protein signalling cascades that boost metabolic and secretory functions.^34^ The interaction between photons and cellular chromophores initiates a series of biochemical events that enhance cellular metabolism and promote the release of bioactive molecules. Photobiomodulation therapy results in increased ATP production and improved cellular metabolism as a result of photon absorption by mitochondrial cytochrome c oxidase. This metabolic stimulation leads to increased protein synthesis, growth factor production and enhanced exosome secretion. In addition, the thermal component of the conditioning process further enhances the therapeutic cascade by improving enzyme activity and cellular transport.^35,36^ Studies have shown that photothermal stimulation can significantly increase exosome and ATP release from PRP preparations and various cell types, without causing harm.^34^ For instance, the study by Mahmood *et al*. demonstrated that thermal stimulation at physiological temperatures increased exosome secretion without cytotoxic effects.^14^ Similarly, Irmak *et al*. found that photoactivated PRP released higher levels of VEGF and maintained its bioactivity for longer periods compared with untreated PRP.^7^

#### Optimization parameters and standardization

**Figure 1:**
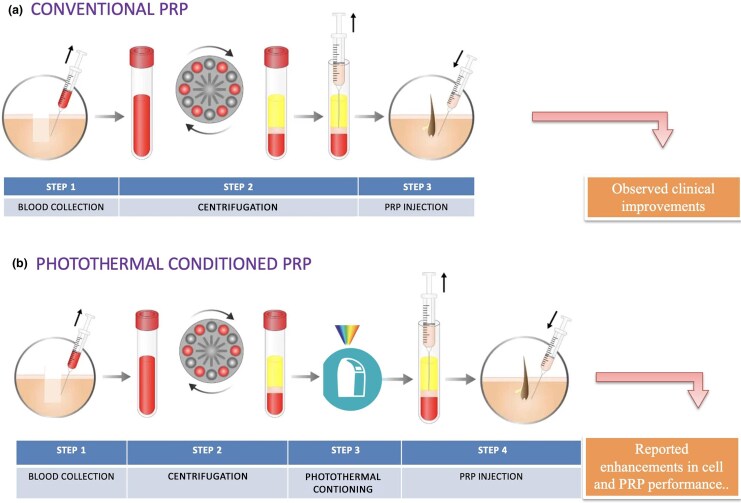
Comparison of conventional and photothermal platelet-rich plasma (PRP) preparation methods for hair restoration. (a) Conventional PRP consists of three main steps: blood collection, centrifugation and direct scalp PRP injection. (b) Photothermal PRP includes an additional conditioning step in which PRP is exposed to controlled thermal and light-based energy prior to injection. This process may regulate cellular activity and increase growth factor release, as suggested by preliminary studies.

Standardization of photothermal conditioning protocols is crucial for reproducible clinical results. However, it should be noted that several critical parameters such as wavelength selection, power density, exposure time and temperature control play a crucial role in the efficacy of photothermal conditioning. In biological tissues, red and near-infrared wavelengths (660–850 nm) have shown optimal penetration depth and biomodulator effects. Therefore, power density should be carefully controlled to achieve therapeutic effects without thermal damage to cellular components.^37,38^ Temperature regulation during the conditioning process not only increases exosome production, but also plays an important role in preserving the viability and function of cellular components. The optimal temperature range of 37–42 °C is considered to enhance therapeutic benefits without compromising cellular integrity. Exposure time usually varies between 5 and 15 min, with 10 min being the most commonly used protocol.^25,39,40^ Factors such as ambient temperature, humidity and storage conditions can significantly affect the efficacy of the treatment. Standard quality control measures for consistent therapeutic efficacy include assessments such as platelet or cell viability, biochemical assays (ATP, growth factors) and exosome quantification (particles, size, cargo content) to ensure consistent therapeutic profiles.^39,40^ Figure 1 compares conventional PRP treatment with photothermal PRP conditioning. While the conventional method involves immediate use after centrifugation, photothermal systems include a controlled energy exposure step. Both workflows use autologous blood products and aim to stimulate follicular regeneration. The additional step in photothermal PRP conditioning may promote mitochondrial activation, ATP release and increased secretion of growth factors. However, the mechanisms and outcomes of photothermal PRP are still under investigation.

### Example of commercial photothermal conditioning system

#### Technical specifications and design

One example of a commercial photothermal conditioning platform is the MCT system (Meta Cell Technology, Barcelona, Spain), which has been designed to standardize PRP photothermal treatment through controlled electromagnetic and thermal energy exposure. The system applies specific wavelengths and controlled temperatures to PRP for a defined duration, typically 10 min. This process has been reported to increase growth factor release and exosome production in a more standard manner.^40^ Precise control of temperature, light exposure and duration are key features in photothermal platforms to ensure reproducibility in clinical applications. The systems also include real-time monitoring of operational parameters such as temperature and light intensity.^15,41^ Photothermal PRP systems often use sterile, biocompatible containers engineered for light transparency and thermal stability. Some platforms include mixing chambers or filters to ensure uniform distribution of PRP and removal of unwanted cellular debris.^12^ Preconditioned PRP with this system for 10 min has been shown to increase the basal concentration of growth factors (e.g. VEGF, IGF-1 and PDGF) by three-fold and intracellular ATP by two-fold, as well as leading to significant exosome enrichment with stable secretion kinetics.^14,15^

#### Clinical practice and regulatory status

At least one photothermal conditioning system has received regulatory approval as a Class IIa medical device in both the US [Food and Drug Administration (FDA)] and Europe (CE), indicating that such platforms are increasingly recognized for clinical use.^41,42^ However, this designation applies to the device itself and does not necessarily extend to all claims or clinical outcomes related to PRP. Advantages of certain commercial systems include easy integration into clinical workflows and the use of standard protocols aimed at reducing operator variability and increasing reproducibility.^12^

#### Summary of comparative outcomes

Photothermal conditioning has been shown to significantly improve the biological activity of PRP by increasing exosome release, upregulating growth factor secretion and stimulating ATP production. Compared with conventional PRP preparations, emerging data suggest that this method can lead to a two- to threefold increase in growth factor levels and up to a fivefold increase in exosome production. Reported clinical results appear promising, with significant improvements in both subjective satisfaction and objective hair measurements. These trends require confirmation in larger studies. As noted in the results, initial safety reports and regulatory approvals support the clinical applicability of photothermal PRP.

## Discussion

Photothermal conditioning of PRP enhances the bioactivity of PRP by regulating cellular metabolism and stimulating the release of essential growth factors and exosomes. This conditioning method may contribute to improved clinical outcomes in hair regeneration. The increased release of exosomes following photothermal activation supports cell–cell communication and regenerative signalling.^6^ It has also been proposed that the composition of exosomes from PRP could serve as a predictive biomarker for the effectiveness of PRP, allowing for more personalized regenerative therapies. For example, the quantification of vesicle-associated microRNAs such as miR-126 has shown promising results in relation to angiogenic response.^43,44^ Increased release of exosomes following photothermal activation supports regenerative signalling and may enable personalization of therapies based on cargo profile.^11^ Meanwhile, exosomes derived from mesenchymal stem cells are rich in bioactive molecules such as proteins, lipids, mRNA and miRNAs, which regulate immune responses, promote angiogenesis, reduce inflammation, and support tissue repair and regeneration.^27^ These properties, which vary based on the cell source, allow exosomes to exist independently or in combination with those from different cell types.

The heterogeneity in exosomal cargos not only provides opportunities, but also challenges for therapeutic applications.^27^ This diversity not only allows for multitarget therapeutic effects, but also complicates standardization and quality control efforts.^45^ Advanced characterization techniques such as flow cytometry, nanoparticle tracking analysis and proteomic profiling are being developed to better understand and standardize exosomal ­preparations.^45,46^

PRP treatment has shown significant improvements in hair regeneration for conditions like androgenetic alopecia and alopecia areata, either as standalone therapy or in conjunction with hair transplantation.^8^ Photothermal PRP conditioning platforms utilize autologous materials that maintain biocompatibility and minimize immunogenic risks compared with synthetic or donor-derived alternatives. Exosomes derived from these preparations are unmodified and reflect the native profile of the cells of origin, potentially reducing the risk of adverse effects such as immune reactions or disease transmission.^8,40^

Studies have shown that photothermal conditioned PRP applications resulting in potentially enhanced outcomes, though direct comparative data are limited. Preconditioning of PRP using polychromatic light for approximately 10 min has been reported to increase the release of essential growth factors and increase ATP production from mitochondria.^7^ These improvements indicate a stronger regenerative effect compared with standard PRP.^47,48^ Patients receiving conditioned PRP show a faster onset of therapeutic effects, greater increases in hair density and longer-lasting ­benefits.^12^

Natural exosomes exhibit excellent therapeutic effects by encapsulating various regulatory proteins, miRNAs, mRNAs and other naturally active substances. However, the effects of exosomes carrying different functional biomolecules on hosts have not been fully understood. Injectable exosome therapies have not been approved for treating hair loss by major regulatory agencies such as the US FDA and the European Medicines Agency.^49^ In contrast, at least one photothermal PRP device has received both FDA and CE regulatory approval as a Class IIa medical device, which may support wider clinical adoption until further evidence of long-term effectiveness is available.^41,42^

Clinical data suggest that the safety profile of photothermal conditioned PRP appears favourable. Adverse events are rare and typically limited to mild injection site reactions, similar to those of traditional PRP treatments. The autologous nature of the treatment eliminates concerns about disease transmission and immunogenic reactions that can occur with allogeneic or xenogeneic therapies.^50,51^ PRP is considered a minimally manipulated autologous product, while the regulatory status of exosomes, particularly those from stem cells is intricate. Photothermal conditioning preserves the autologous nature of PRP without genetic or chemical modifications, potentially aligning better with existing regulatory frameworks.^52^ Practitioners should ensure compliance with local medical device regulations and ethical guidelines when using these protocols. Preliminary reports indicate that there are no significant long-term adverse effects associated with PRP in clinical use. However, ongoing monitoring is essential to establish comprehensive safety profiles for these new technologies. Data from our observations, pilot studies and case reports support the effectiveness of photothermally enhanced PRP in hair restoration.^8,53^ Patients receiving photothermal conditioned PRP showed improved hair density, increased hair shaft thickness, enhanced scalp vascularization and reduced inflammatory markers.^53^ Hernández Sanz and Pinto reported superior outcomes in facial skin rejuvenation with photothermally activated PRP.^12^ This suggests broader applications for this technology beyond hair restoration. The versatility of photothermal conditioning platforms suggests potential applicability beyond hair restoration, including wound healing, wound modulation and other tissue regeneration settings. However, clinical evidence in these areas remains limited and exploratory.

In conditions like alopecia where inflammatory dysregulation (e.g. elevated interleukin-6, tumour necrosis factor-α, node-like receptors) hinders hair follicular regeneration, MCT-augmented PRP treatment may stabilize the local immune environment, prolong the anagen phase and enhance follicular neogenesis.^7^ A systematic review has noted that PRP growth factors regulate inflammation for immune-mediated alopecias (including alopecia areata and cicatricial forms) and may be useful in conditions where standard treatments are limited.^54^ Patient-reported outcome measures consistently show high satisfaction rates with ­photothermal-enhanced PRP treatments. Improvements in quality of life assessments, psychological wellbeing and self-esteem after treatment highlight the broader impact of successful hair restoration treatment than just clinical success.^55,56^ In a retrospective study, Hetz *et al*.^17^ reported a mean patient satisfaction score of 7.3/10 and a likelihood of recommending PRP of 8/10. Other approaches like platelet activation with calcium chloride or thrombin, addition of synthetic growth factors, combining PRP with laser therapy, pharmacological methods or stem cells are also being explored. While these methods show promise, photothermal enhancement offers distinct advantages, including the preservation of autologous status, increased exosome production and long-term bioactivity.^29^ Chemical activation methods, while effective in promoting platelet degranulation, may alter the natural balance of bioactive molecules and potentially introduce foreign substances that could potentially trigger immune responses.^57^ Photothermal conditioning, in contrast, preserves the integrity of the autologous system by enhancing natural cellular processes without chemical changes.^7,11^ Combination treatments using photothermally enhanced PRP with other treatment modalities are being investigated. Preliminary studies suggest that combining conditioned PRP with low-level laser therapy, microneedling or topical growth factors has synergistic effects. A randomized study combining microneedling and low-level laser therapy reported significant improvements in hair count and density compared with PRP alone.^58^ A study analysing PRP + microneedling found that the combination application was better tolerated and safer. It was also observed that the combination resulted in higher patient satisfaction and an average increase of ∼19 hairs per cm².^59^ These multimodal approaches may provide enhanced therapeutic benefits while minimizing individual treatment limitations.

Currently, there is a lack of clinical data, and large-scale studies are needed to address this issue. To provide definitive evidence on the efficacy and safety of photothermally enhanced PRP in hair restoration, planned multicentre randomized controlled trials should include diverse patient populations, standardized outcome measures and extended follow-up periods to establish evidence-based treatment protocols.^25^ Additionally, there is variability in clinical responses among individuals, necessitating research on predictive biomarkers and personalized treatment approaches.^11^ Specific conditions, such as genetic polymorphisms affecting growth factor production, platelet function and hair follicle responsiveness, may require additional research to optimize patient selection and treatment protocols. One area of future research will focus on determining the optimal light wavelengths, temperatures and exposure times to establish standardized protocols. Extended follow-up studies should also be conducted to evaluate long-term efficacy. Table 2 summarizes the common limitations of current PRP and photothermal PRP research.

**Table 2:** Summary of common limitations in current platelet-rich plasma (PRP) and photothermal PRP literature

Category	Challenge	Consequence
Research design	Small sample sizes and single-site studies	Weakens statistical strength and generalizability
Protocol variability	Variability in PRP processing, activation protocols and photothermal settings	Hinders standardization and between-study analysis
Evaluation criteria	Nonuniform use of objective tools (e.g. trichoscopy, hair density)	Obstacles to intertrial effectiveness evaluation
Follow-up duration	Majority of trials restricted to ≤12 months	Long-term durability of treatment remains unclear
Control groups	Lack of comparator or sham groups in many studies	Hard to determine treatment-specific effect
Patient stratification	Insufficient participant stratification by age, gender, hair loss grade or genetic profile	Impedes tailored treatment planning
Regulatory clarity	Nonstandardized categorization of PRP/exome therapies	Unclear clinical adoption and regulatory adherence
Mechanistic evidence	Limited molecular characterization of inflammatory markers, miRNA or exosome profiling	Constrains knowledge of therapeutic mechanisms

miRNA, microRNA; NA, not applicable; t-PRP, temperature-controlled PRP.

Advanced imaging technologies, including high-resolution ultrasound and optical coherence tomography, are being developed to provide real-time monitoring of hair follicle responses to treatment. These tools will enable more precise assessment of treatment efficacy and optimization of individual therapeutic protocols based on individual patient responses.^60^

Artificial intelligence and machine learning algorithms are being developed to analyse treatment outcomes and predict optimal protocols for individual patients. This technology may enable personalized treatment approach based on patient characteristics, medical history and treatment response patterns.^61^ Nanotechnology applications in exosome engineering and targeted delivery systems represent emerging areas of research that could further enhance the therapeutic potential of photothermally conditioned PRP. Controlled release systems and targeted delivery mechanisms could improve the precision and duration of therapeutic effects.^62,63^

While initial findings suggest that photothermal conditioning enhances the biological activity of PRP, the current evidence base is limited by significant methodological variability. Many studies lack standardization in PRP preparation protocols, including differences in centrifugation speeds, thermal exposure times and light transmission parameters. This heterogeneity makes direct comparisons difficult and impairs reproducibility. Furthermore, most clinical studies to date have been small-scale pilot studies or case series with limited sample sizes, typically fewer than 20 participants. Control groups are often lacking, and objective measurements such as quantitative assays for exosomes, cytokines or ATP are inconsistently reported. The short follow-up period further limits conclusions about the durability of clinical outcomes.

Overall, small sample sizes, the lack of standard thermal protocols and limited biochemical profiling limit the generalizability of the current findings. Larger, randomized controlled trials with consistent methodology and detailed quantitative analysis of PRP components are urgently needed. Table 3 summarizes the limited number of studies comparing conventional and photothermal PRP, including methodology, results and noted gaps.

**Table 3:** Quantitative studies on photothermal conditioned platelet-rich plasma (PRP)

Design type	Sample size	Comparison conducted	Main results	Limitations	Reference
*In vitro*, imaging	NA	Exosome secretion at different temperatures	Higher temperatures increase the frequency of exosome release	Not PRP-based; no growth factor data	^14^
Prospective clinical	7	Photothermal PRP and conventional PRP	Improvement in skin laxity and satisfaction in the photothermal PRP group	No biochemical analysis; limited to aesthetics	^12^
Prospective clinical	10	PRP and placebo (hair regeneration)	Increased hair density in the PRP group	No photothermal comparison	^53^
Commercial report	NA	PRP pre- and post- photothermal	Claims 3× growth factor and 2× ATP increase	Not peer-reviewed; lacks methodological details	^15^
Experimental (clinical)	40	t-PRP vs. conventional PRP (no activator)	PDGF and VEGF levels significantly higher in thermally activated PRP	The thermal protocol included heating at 56 °C for 30 min, followed by a freeze–thaw cycle and a final incubation at 37 °C for 15 min to produce activated t-PRP	^13^
Experimental (*ex vivo*)	16	Before and after PRP thermal conditioning	EGF ↑98.8%, bFGF ↑84.8%, VEGF ↑43.2%; no change in PDGF	Semi-quantitative analysis; short conditioning time	^64^

bFGF, basic fibroblast growth factor; EGF, epidermal growth factor; NA, not applicable; PDGF, platelet-derived growth factor; t-PRP, temperature-controlled PRP; VEGF, vascular endothelial growth factor.

## Conclusion

Photothermal conditioning of PRP is a novel strategy that could boost the regenerative capabilities of autologous blood products by modulating cellular and molecular components. Although initial data are encouraging, the field is still in its early stages and key challenges remain, including a lack of standardized protocols, limited mechanistic understanding and the need for larger, controlled clinical trials. As an additive-free and autologous approach, photothermal PRP may offer practical and safety advantages. However, further research is needed to determine its role relative to other PRP development strategies within the field of regenerative medicine.

## Data Availability

All data supporting data are included in the article.
